# A Fuzzy-Based Model to Detect Hotspots of Air Pollutants During Heatwaves in Urban Settlements

**DOI:** 10.3390/s25072160

**Published:** 2025-03-28

**Authors:** Barbara Cardone, Ferdinando Di Martino, Cristiano Mauriello, Vittorio Miraglia

**Affiliations:** 1 Department of Architecture, University of Naples Federico II, Via Toledo 402, 80134 Napoli, Italy; b.cardone@unina.it (B.C.); cristiano.mauriello@unina.it (C.M.); vittorio.miraglia@unina.it (V.M.); 2 Center for Interdepartmental Research “Alberto Calza Bini”, University of Naples Federico II, Via Toledo 402, 80134 Napoli, Italy

**Keywords:** air pollution, heatwave, hotspot, fuzzy partition, spatial interpolation

## Abstract

High concentrations of pollutants in urban areas generate cardiovascular and respiratory problems in citizens; these are aggravated by the persistence of summer heatwaves. For this reason, in this research, we propose a fuzzy-based method for detecting air pollutant hotspots and determining critical urban areas for air pollution during heatwaves. After acquiring the pollutant concentration values recorded by monitoring stations during heatwaves, a spatial interpolation method is applied to obtain the distribution of the pollutant concentration during heatwaves and, subsequently, a fuzzification process is performed to determine urban hotspots in which the pollutant concentration assumes critical values. Finally, the critical urban areas are determined, consisting of the areas within hotspots with a high population density exposed to health risks. The method was implemented in a GIS platform and tested on an urban study area in the Lombardy region, Italy, to determine the urban areas with high criticality during the heatwaves that occurred in the summer months of 2024. The test results show that the method can provide valid support for decision makers and local administrators when evaluating which urban areas are most critical for the population due to the high rate of air pollution during heatwaves.

## 1. Introduction

Air pollution is a significant risk factor for the health of people living in urban agglomerations. It is produced by human activities that encourage the dispersion of gases and extremely fine particles in the atmosphere, such as driving, using energy-producing facilities, and industrial operations.

Airborne pollutants are linked to a variety of diseases, the most common of which are immune system, cardiovascular, and respiratory illnesses. These environmental pollutants can trigger inflammatory reactions and worsen pre-existing problems, which can lead to the development and advancement of several chronic illnesses [1,2,3,4].

Two of the more significant environmental stressors on health are exposure to air pollution and exposure to extreme temperatures [5]; the combined effects of the two phenomena on citizens’ health have been studied elsewhere [6].

A significant correlation was highlighted [7] between the increase in allergic respiratory diseases, such as rhinitis and bronchial asthma, and environmental and climatic risk factors such as air pollution and increased average temperatures. Recent studies have shown that the main determining factors for the onset of respiratory problems among urban residents are the increase in temperatures and exposure to high levels of air pollution. A collection of the results of these studies is included in [8].

In order to identify the urban air pollution hotspots created during heatwaves and identify which urban regions are affected, we present a novel approach for identifying air pollution hotspots in urban settlements.

In this research, we propose a new method for detecting air pollution hotspots in urban settlements generated during heatwaves and for determining which urban areas included in hotspots are more critical due to a greater risk to the health of vulnerable citizens.

Following [9], a pre-analysis is conducted to identify the time steps during the investigated period when the phenomenon occurred in the study area. For this purpose, a preprocessing phase is set up, in which the time intervals when heatwaves occurred (time frames) are determined for the entire investigated period.

For each time frame, daily data on the concentration of the air pollutants measured by a set of Monitoring Central Units (MCUs) are acquired and a spatial interpolation model is used to obtain the distribution of the pollutant throughout the study area. Using a fuzzy-based classification approach, urban areas with high concentrations of pollutants are detected as hotspots, and critical urban areas located in hotspots with a high density of disadvantaged residents who are exposed to the risk of worsening respiratory and cardiovascular diseases are identified.

High replicability is provided by the model because it does not require high-resolution local datasets, but only MCU readings, which are used to assess the spatial distribution of pollutant concentrations and population census data; these are then used to evaluate the density of the population of disadvantaged residents. The model can, therefore, be replicated in another urban context, requiring only the measurements recorded by the MCUs located in the study area and the census data provided by the population census bodies.

Additionally, the model is user-friendly, as it does not require many parameters to be set; additionally, it uses a fuzzification approach that makes it easier for the user to assess the possible serious health risks posed to residents in hotspot locations during heatwaves.

The model represents a helpful tool for supporting local decision makers in determining which urban areas are most at risk and in implementing design strategies and precautions to safeguard public health.

This research represents a significant advancement in detecting and managing environmental risks in urban settings, with important implications for public health and urban planning:

- Identification of critical air pollution areas during heatwaves: this study proposes an innovative method to detect urban air pollution hotspots during heatwaves, identifying the areas most at risk in terms of the health of vulnerable populations.
- Methodological approach based on temporal and spatial analysis: the method includes a preliminary preprocessing phase to identify heatwave periods and collect daily data on air pollutant concentrations, normalizing the values according to the duration of the event.
- Spatial interpolation to model pollutant distribution: using spatial interpolation techniques, the model constructs a detailed representation of pollutant distribution during critical periods.
- Classification of urban areas through fuzzification: a fuzzification process classifies urban areas based on their level of health risk, highlighting the most critical hotspots.
- Generation of criticality maps for public health protection: the method produces risk maps that identify urban areas with higher densities of vulnerable populations, supporting the planning of targeted interventions.
- High transferability and ease of implementation: the model stands out for its high transferability, requiring only a limited amount of data (daily measurements from monitoring stations and demographic data), making it easily applicable to different urban contexts.
- Decision support for urban planning and environmental policies: by providing a simple yet effective tool, this method can assist local authorities in designing air pollution mitigation strategies and protecting public health.

## 2. Related Research

In the last decade, research has addressed the detection of the most critical urban areas, which are more sensitive to the dispersion of pollutants, as well as those that are, on the other hand, more resilient.

The first line of research was focused on the detection of urban settlements where ventilation was most impeded. A method for detecting ventilation zones in urban settlements, taking into account the compactness of the urban form, the average height of the buildings, and the typology of the prevalent road model, was proposed in [10]. The method was adopted to determine which urban areas were less ventilated and, therefore, more at risk in the presence of air pollutants. A GIS-based framework aimed at identifying urban areas with lower ventilation potential, which are more at risk in the presence of high concentrations of air pollutants, was implemented in [11].

Other research has aimed to analyze the distribution of the pollutants in the urban fabric to evaluate the types of urban patterns and soils in which the concentration of the pollutants was particularly high. A modelling study, based on data acquired from mobile stations, of the spatial distribution of air pollutants in the metropolitan area of Kano, Nigeria, was carried out in [12]. This study highlighted that the areas most exposed to air pollution are urban patterns, and mainly industrial and commercial areas. An air pollution urban hotspot detection method based on the spatiotemporal trend of air pollutants was proposed in [13]. To detect hotspots, three parameters were taken into account: the temporal frequency of values exceeding the threshold, given by the percentage of days in which the thresholds are exceeded, the level at which the threshold values are exceeded, and the number of consecutive days of exceeding. The method has been applied to detect hotspots in Delhi city, India, considering PM_2.5_ concentration measurements in the summer months of the years from 2018 to 2021.

A hierarchical clustering method is applied in [14] to detect urban air pollutant hotspots using data acquired from mobile stations installed on trash-trucks driven along the urban streets of Cambridge, Massachusetts. The analysis of the detected hotspots allowed the researchers to determine which were the main sources of pollution.

The need for many measurements of the concentration of the pollutants makes it difficult to carry out these methods in different urban settlements. In fact, to apply the cluster-based models of the spatial distribution of pollutants proposed in [13,14] to other urban contexts, it is necessary to make use of a significant amount of measurements detected by mobile stations, which would require prolonged and expensive preprocessing activity.

Furthermore, these studies do not take into account other types of environmental and climatic risks that can further increase the phenomenon and the risk to the health of the resident population. In particular, emerging climatic scenarios, such as the presence of increasingly intense summer heatwaves, can aggravate both the concentration of air pollutants, for example, due to the absence of ventilated and current areas during periods of heatwaves, and the health problems associated with the cardio-respiratory system.

Finally, socio-economic conditions can also affect the detection of critical areas during heatwaves and high concentrations of air pollutants. An emotion detection method aimed to analyze the emotional categories expressed by citizens during heatwaves; the study highlighted that the areas where unpleasant emotions prevail and citizens’ discomfort is felt most strongly are urban areas with a high-density population of poor and vulnerable people [15].

Several factors can increase or reduce the concentration of pollutants in the atmosphere. These factors are mainly local, connected both to the morphology of the urban settlement (the presence or absence of ventilation channels, proximity to the sea, density of buildings, presence of green wooded areas, etc.) and to specific anthropogenic factors such as the presence of industrial plants, the excessive use of air conditioners, and road traffic. Furthermore, especially in dense urban settlements, to carry out an analysis of correlations between the concentration of air pollutants and the increase in temperatures, it is necessary to consider multiple parameters that characterize the study area.

A study of the distribution of air pollutants in urban settlements to determine critical urban areas was performed in [16]. The authors propose a method in which the urban settlement is partitioned into Thiessen polygons based on the daily concentration of pollutants measured by fixed air quality monitoring stations located in the urban settlement during a heatwave. They tested the method in the city of Bologna in Italy. This method is computationally fast; however, the partitioning of the area into Thiessen polygons can provide a poor, inaccurate spatial distribution of the pollutant concentration. This can affect the spatial accuracy of the detected hotspots.

The kernel density method is applied in [17] to assess the spatial distribution of pollutant concentrations and to detect hotspots. However, this method does not apply to the detection of air pollutant hotspots from fixed air quality monitoring stations, as it requires measurement points with a high spatial resolution.

Forecasting models are proposed by some authors to assess the air pollutant spatial distribution. Fuzzy time series [18,19] and fuzzy Markov chains [20] are applied to predict the daily air pollution indices. The critical point of these models is that the forecasting accuracy depends on the number of partitions of the universe of discourse of the time series data used in the models.

To increase the forecasting accuracy and the spatial accuracy of the air pollutant hotspots, deep learning techniques based on long short-term memory networks have recently been proposed in [21,22,23,24] to predict the spatial distribution of air pollutant concentrations. Although they increase the accuracy of predictions compared to traditional models, these models are computationally expensive and require metaheuristic approaches for parameter setting [25].

The proposed method is discussed in detail in Section 3, where the case study is presented. The results of the experimental tests are shown and discussed in Section 4. Concluding remarks are given in Section 5.

## 3. Materials and Methods

In this study, a method for detecting hotspots in the study area was tested, in which critical values of an air pollutant are measured during heatwaves that occurred in the summer months.

In the investigation period, the time frames in which heatwaves were recorded in the study area are determined. Following [26], a heatwave time frame is detected by the presence of a heat index value higher than 32 °C for at least three consecutive days, where the heat index is a measure of perceived temperature defined by the USA National Weather Service, combining daily air temperature and relative humidity [27].

Subsequently, for each of these time frames, the daily measurements of the air pollutant concentration recorded by the monitoring central units (MCUs) located in the study area are extracted. For each time frame, a spatial interpolation algorithm is used to model the distribution of the pollutant concentration in the study area. Upon completion of the construction of the pollutant distribution for each time frame, raster data are generated, in which each cell is assigned the average value of the pollutant concentration in the individual time frames, weighted by the duration of the time frame.

Then, a fuzzification process is performed in which each cell is classified based on the level of concentration of the pollutant. This classification is accomplished by building a fuzzy partition of the pollutant concentration values and assigning to the cell the label of the fuzzy set to which it belongs with the highest degree of membership.

Finally, an aggregation process is performed, in which the contiguous cells assigned to the same fuzzy sets are merged to form polygons on the map. The result of this process is a thematic map in which the urban areas classified with high danger levels constitute hotspots where the population is most at risk.

Formally, let d_1_, …, d_n_ be the durations in number of consecutive days of the n time frames in which heatwaves occurred during the analyzed period. $m_{i}^{j}$ denotes the average daily peak of pollutant concentration recorded by the jth MCU during the ith time frame. The set M_i_ = {$m_{i}^{1},m_{i}^{2},\ldots ,m_{i}^{N}$} represents the set of the average daily peak of pollutant concentration registered by all the N MCUs located in the study area.

The result of the spatial interpolation process applied to the set of data points M_i_ is a raster dataset in which $m_{i}\left(h,k\right)$ denotes the interpolated value in the (h,k)th cell covering the study area.

At the end of the spatial interpolation process performed for all n time frames, a raster is constructed in which each cell is assigned the weighted average of the interpolated values, where the weight is the duration of the time frame. The value assigned to the (h,k)th cell is given by:

(1) $$v\left(h,k\right)=\frac{\sum_{i=1}^{n}m_{i}\left(h,k\right)d_{i}}{\sum_{i=1}^{n}d_{i}}.$$

To perform the fuzzification process, a fuzzy partition of the concentration of air pollutants is created, based on the severity of the effect on people’s health. The fuzzy partition is constructed using fuzzy numbers and respecting the Ruspini condition, a constraint that establishes that the sum of the membership degrees of an element to the fuzzy sets of the fuzzy partition is equal to 1 [28]. The use of fuzzy partitions composed of fuzzy numbers facilitates the usability of the framework because it models human approximate reasoning more closely than the use of other types of fuzzy sets. The Ruspini constraint ensures that a cell belongs to the fuzzy sets of the fuzzy partition with a cumulative membership degree equal to one; this means that the cell is assigned to the union of all fuzzy sets of the fuzzy partition with certainty.

The fuzzification process assigns to the (h,k)th cell the label of the fuzzy set to which the value $v\left(h,k\right)$ belongs with the greatest membership degree.

Then, all contiguous cells belonging to the same fuzzy set are merged to form a polygon on the map. Polygons classified with high danger levels are considered hotspots.

In order to determine the most critical areas, an assessment of the population most exposed to health risk residing in each hotspot is carried out. This is achieved by acquiring population census data and calculating the estimate of the exposed population residing in the hotspot.

**Figure 1:**
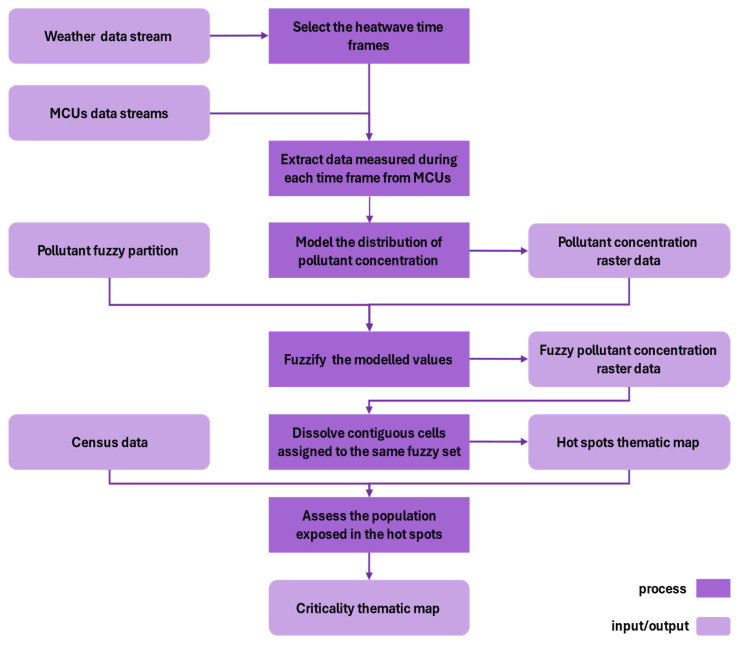
Flow diagram of the proposed method.

The result of the process is a thematic map in which the hotspots are classified based on the criticality for residents exposed to health risks. The method is schematized in Figure 1.

The component labelled *Model the distribution of pollutant concentration* executes a spatial interpolation process for each time frame; then, it creates a raster called *Pollutant concentration raster data* using Formula (1). 

The component labeled *Fuzzify the modeled values* employs a fuzzy partition of the pollutant concentration (*Pollutant fuzzy partition*) to fuzzify the modeled concentration values. Specifically, it transforms the concentration data into fuzzy values by mapping each pixel of the raster to the fuzzy set that exhibits the highest degree of membership (*Fuzzy pollutant concentration raster data*). The output is a raster where each cell is assigned the label of the fuzzy set corresponding to the highest degree of membership, based on the fuzzy partition of pollutant concentration.

The functional component *Assess the population exposed in the hotspot* leverages demographic data from population censuses to identify individuals at heightened health risk within each previously delineated hotspot. By integrating spatially explicit environmental data with the population distribution, this component quantifies the degree of exposure for various demographic groups within these hotspots. The resulting output is a *Criticality thematic map* wherein each hotspot is stratified according to the proportion of the resident population exposed to the environmental hazard. These hotspots are subsequently classified into distinct criticality levels, reflecting the intensity of exposure and the potential health impact on the affected population. This approach allows for a targeted identification of areas in greatest need of intervention, providing a valuable tool for public health policy and resource allocation.

### The Case Study

The framework was tested on a study area extending to the Lombardy region in Italy, to detect the most critical areas for the presence of high concentrations of nitrogen dioxide (NO_2_) during heatwaves in the year 2024.

Nitrogen dioxide is formed in the atmosphere by the oxidation of nitrogen monoxide (NO), which is mainly emitted by anthropogenic sources due to industrial combustion processes, heating systems, and vehicular traffic, as well as through production processes without combustion, such as the use of nitrogen fertilizers in agriculture.

It causes alterations in lung function in humans, generating chronic bronchitis, asthma, and pulmonary emphysema. Those most at risk are children and people already affected by respiratory diseases (asthmatics), as well as those living near roads with high traffic density due to long-term exposure.

The air quality sensor network consists of local monitoring stations equipped with automatic analyzers, which deliver continuous data at regular intervals. The pollutants that were continuously monitored include NO_X_, SO_2_, CO, O_3_, PM_10_, PM_2.5,_ and benzene. The specific pollutants to be detected vary depending on the environmental context in which the monitoring is conducted; consequently, not all stations are equipped with the same analytical instrumentation. Regional monitoring stations are strategically located across the territory in accordance with population density and land characteristics, adhering to the guidelines outlined in Italian Legislative Decree 155/2010. The station placement takes into account key factors such as the emission load, orographic features, meteorological–climatic conditions, and the degree of urbanization of the area.

The equipment installed in the air quality monitoring stations, which operate 24 h per day, 365 days of the year, is therefore constantly subjected to checks, aimed precisely at ensuring the proper functioning of the equipment over time and the reliability of the data produced. To ensure the quality of the data, in addition to these periodic checks and maintenance interventions, quality assurance procedures are undertaken to ensure the correct functioning of the network and the quality of the measurements taken by the MCUs. Periodically, blind test activities are also carried out to verify the proper functioning of the sensors and the reliability of the measurements. The number of MCUs depends mainly on the urban structure, the distribution of the resident population and the state of air quality: the density of MCUs in areas with low detected concentrations of pollutants is lower than that in areas with high population and building densities. The distribution of MCUs ensures an accurate estimate of air quality both in more critical areas, such as busy urban areas, and in areas far from direct pollution sources.

The surface of Lombardy is divided almost equally between plains (which represent about 47% of the territory) and mountainous areas (which represent 41%). The remaining 12% of the region is hilly.

Lombardy is the Italian region with the highest population density and with the most pronounced presence and density of industrial infrastructure. Air pollution is among the issues that have most worried local administrators, who, for several years, have promoted policies to reduce pollutants with the aim of respecting the limit values of the annual averages. In particular, in recent years, the average annual values of NO_2_ recorded by the MCUs located in the Lombardy region have been below the limit value of 40 μg/m^3^.

**Figure 2:**
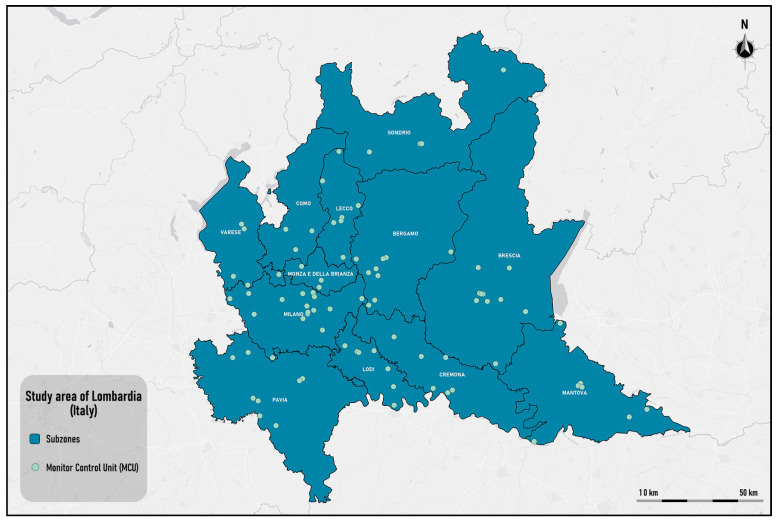
The study area: the Lombardy region.

However, in several periods of the year, hourly values higher than the hourly threshold of 200 μg/m^3^ defined by the World Health Organization have been recorded, especially in dense urban settlements. The study area is shown in Figure 2.

The Lombardia Region is divided into twelve provinces: Bergamo, Brescia, Como, Cremona, Lecco, Lodi, Mantua, Milan, Monza and Brianza, Pavia, Sondrio, and Varese. There are 162 MCUs in total, of which 85, shown on the map in Figure 2, monitor the NO_2_ parameter. The largest number of MCUs is concentrated in the provinces of Milan (16) while the province of Sondrio has the lowest number of MCUs in its territory (4).

**Figure 3:**
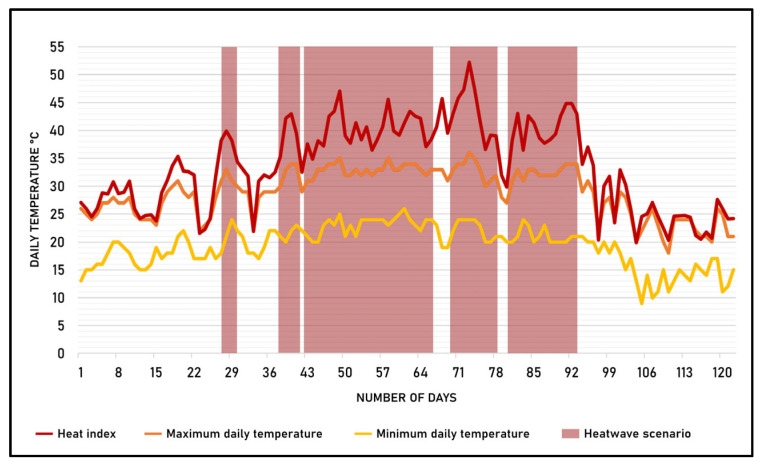
Daily temperature parameter trends during the period 1 June to 30 September 2024.

The analyzed time period extends from 1 June to 30 September 2024, for a total of 123 consecutive natural days. Figure 3 shows the trend of the indicators needed to estimate the heatwave scenario: the daily maximum temperature, the daily minimum temperature, and the heat index.

The figure shows that, during the analyzed period, five heatwave scenarios with different durations occurred. Table 1 shows the summary data of the scenarios.

The heatwave scenario with the longest duration is the third scenario, with a total duration of 25 consecutive natural days.

**Figure 4:**
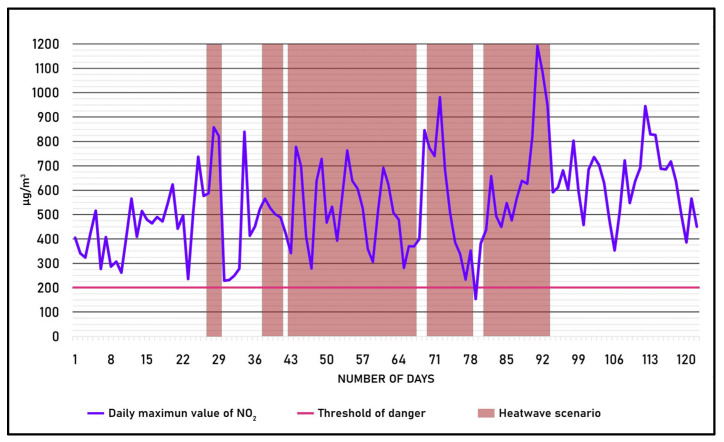
Trend of the maximum value of NO_2_ during the period 1 June to 30 September 2024.

By analyzing the data of the NO_2_ parameter, obtained from an MCU in the study area located in the province of Milano, the trend of the parameter was calculated for the entire period by comparing it with the recorded heatwave scenarios; the trend is shown in Figure 4.

The graph clearly shows that the NO_2_ parameter records its highest values in conjunction with the occurrence of most heatwave scenarios.

The spatial distribution of the NO_2_ parameter was performed through a spatial interpolation process using the Kriging model. Starting from the daily readings of the NO_2_ parameter in the 85 stations of the study area, for each heatwave scenario, the daily peaks were considered as input data for the spatial interpolation process. The cell size is fixed to 50 × 50 m, and the exponential semi-variogram model is applied to assess the distribution of the NO_2_ parameter.

The semivariogram function was selected by analyzing the distribution of variance obtained using different ordinary and universal semivariogram models. The exponential semivariogram model was selected because it provided the optimal distribution of variance with the smallest spatial distribution of the standard deviation. We did not consider other Kriging models, such as empirical Bayesian Kriging, because of their slowness; in fact, using them would have undermined the portability and the scalability of our framework, since their execution speed decreases exponentially as the size of the sample dataset increases.

This spatial interpolation process was performed for all five heatwave scenarios; subsequently, using a map algebra process, a raster was created through the weighted average of the Kriging of the individual scenarios, in which the weights are attributed based on the duration of each scenario, expressed in numbers of consecutive days, as reported in Table 1.

The resultant raster data assessing the spatial distribution of NO_2_ are subsequently classified according to the values reported in the Italian national legislative decree n° 155 of 2010, shown in Table 2.

The fuzzy partition was created by referring to the Italian national legislative decree n° 155 of 2010, which establishes, as thresholds for the health of citizens, two hourly thresholds of NO_2_ concentrations, a minimum threshold of 100 μg/m^3^ and a maximum of 140 μg/m^3^, equal to 50% and 70%, respectively, of the hourly limit value of 200 μg/m^3^ established by the World Health Organization [29]. Furthermore, the legislative decree establishes a highly critical alarm threshold of 400 μg/m^3^, equal to two times the limit value.

**Figure 5:**
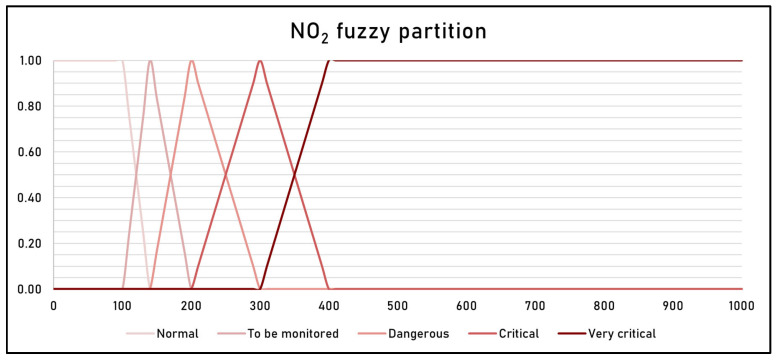
NO_2_ concentration fuzzy partition.

Figure 5 shows the fuzzy partition. It is composed of five fuzzy sets, labeled *Normal*, *To be monitored*, *Dangerous*, *Critical* and *Very critical*. Each fuzzy set is constructed as a triangular fuzzy number.

The population exposed to risk is constituted by the disadvantaged resident density [26], measured as the number of disadvantaged residents per square kilometer, where the number of disadvantaged residents is given by the number of children under 6 years of age and elderly people over 74 years of age.

To calculate the index, the 2024 population census data provided by the Italian Institute of Statistics (ISTAT) were acquired; the ISTAT is the national body that periodically carries out the population census by census area throughout the Italian territory.

Following [26], the disadvantaged resident density is considered critical if is equal to or greater than 5000 disadvantaged residents per square kilometer. The criticality map is built by determining the critical areas inside the hotspots. Table 3 shows the classification of the disadvantaged population density.

The model was implemented in the GIS platform ESRI ArcGIS Pro 3.4 suite using the ESRI ArcGIS Pro 3.4 Python library.

The test results are presented and discussed in the next section.

## 4. Results and Discussion

**Figure 6:**
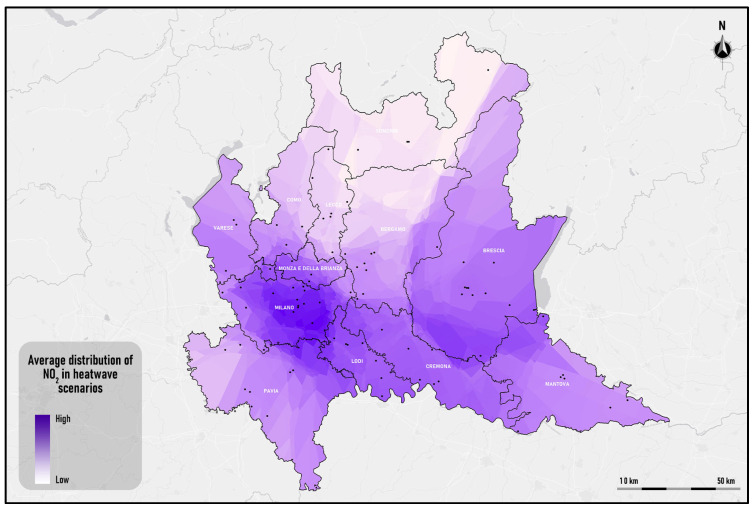
Average distribution of NO_2_ in heatwave scenarios. The black dots are the locations of the MCUs.

The result of the spatial interpolation process is a raster dataset representing the spatial distribution of the mean values of the NO_2_ concentration peaks during the investigation period. This outcome is shown in Figure 6.

The raster dataset of the average spatial distribution of NO_2_ was classified into five classes, based on the values in Table 3 and using the fuzzy partition in Figure 5.

After completing the fuzzification of the distribution of NO_2_ and having performed the dissolution of contiguous cells belonging to the same fuzzy set, the thematic map of the hotspots detected is obtained, where the areas classified as very critical are identified as hotspots.

**Figure 7:**
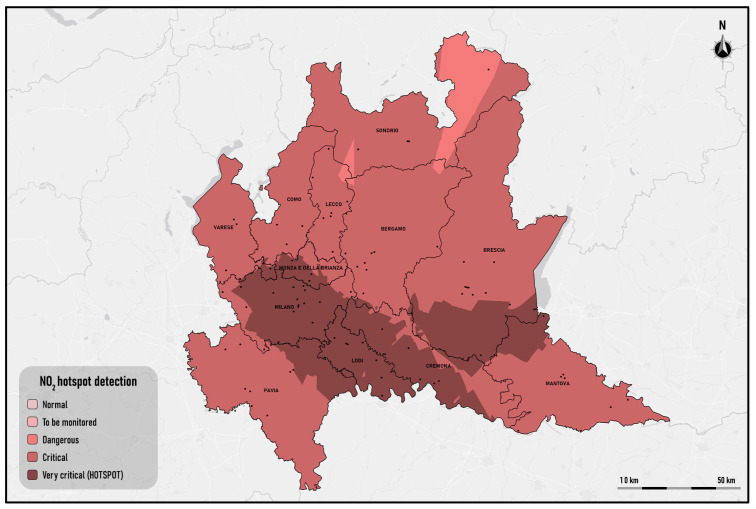
NO_2_ hotspot detection in heatwave scenarios.

The thematic map of the hotspots is shown in Figure 7.

On the map, there are two hotspots: sites in the central–southern area of the region. The two hotspots are located in the areas where Arpa Lombardia, the regional environmental protection body, has detected extremely high concentration levels of NO_2_ during the investigation period; they include extensive urban areas of the provinces of Milan, Monza, Lodi, Cremona and Brescia (https://www.arpalombardia.it/agenda/notizie/2025/qualita-dell-aria-dati-certificati-per-il-2024/) (accessed on 1 December 2024).

The first extends over almost the entire area of the province of Milan and Cremona, completely including the province of Lodi.

Compared to the previous one, the second hotspot has a smaller surface extension, and it is in the immediate vicinity of the former: specifically, in the southern area of the province of Brescia, on the border with the district of Mantova.

**Figure 8:**
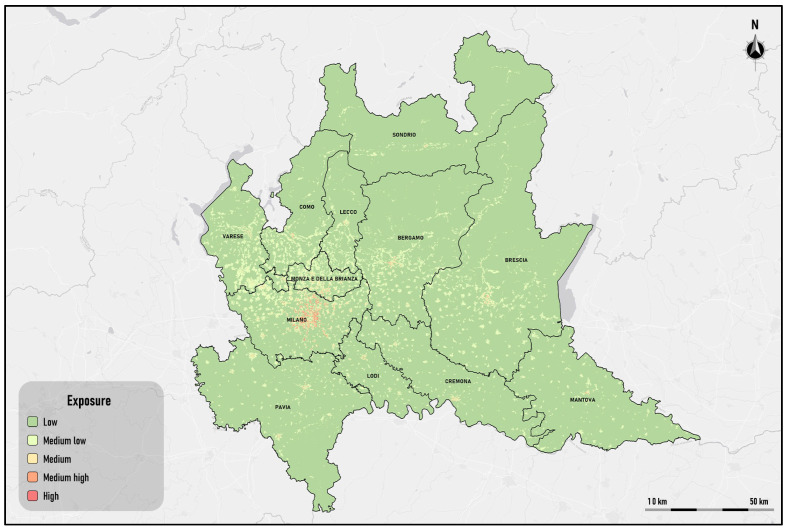
Exposure: classification of disadvantaged population density.

Figure 8 shows the distribution of disadvantaged population density in the study area. The thematic map of is obtained considering the classification of disadvantaged population density shown in Table 3.

The areas with the highest density are concentrated in the provinces of Milan, Monza and Brianza, Bergamo, and Brescia. The northern provinces have the lowest density of disadvantaged populations.

These outcomes are in line with the results of the analysis performed in [30], where both a network of ground-based sensors and satellite images were used to obtain an accurate NO_2_ spatial distribution over an area including the metropolitan city of Milan. The most critical zones were the ones including the city of Milan and the areas of the provinces of Monza and Brianza.

**Figure 9:**
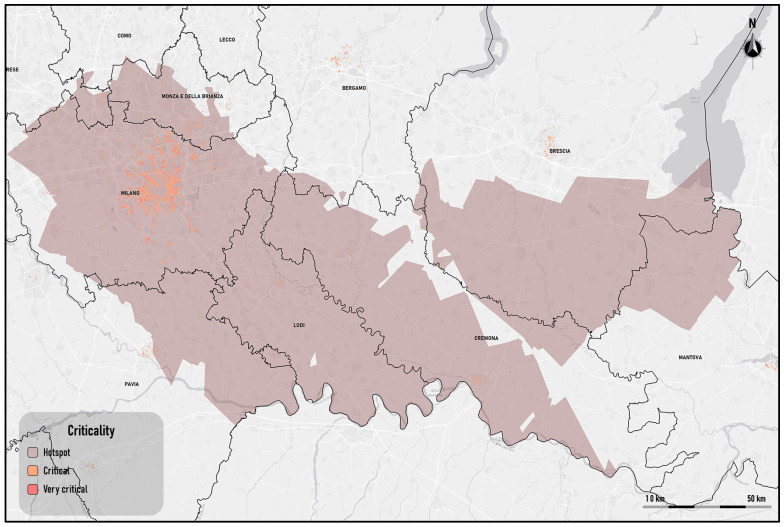
Thematic map of NO_2_ criticality by disadvantaged population density.

Starting from the detection of NO_2_ hotspots and the assessment of the exposed population, the most critical areas for the population exposed to NO_2_ emissions have been identified (Figure 9).

The hotspot located in the east does not represent a critical area; meanwhile, the hotspot with the largest extension encompasses critical and very critical areas near the city of Milan, in the southern area of the province of Monza and Brianza, and in correspondence with the densest urban aggregates of the provinces of Lodi and Cremona.

These four cities are all characterized by high vehicular traffic and a city fabric characterized by the presence of industrial complexes, both factors that significantly affect the concentration of pollutants.

The city of Brescia records a different behavior compared to other cities. Its city center is characterized by some critical areas but does not fall within the identified hotspot. This mainly because of the location of production facilities and the greater concentration of vehicular traffic located in the south-east area of the province, where one of the two hotspots is located.

These results agree with the findings of the studies carried out in [31] relating to the analysis of the trends of the concentrations of air pollutants in the Lombardy Region. These studies were based on the measurements of the MCUs, which highlight the presence of higher concentrations of NO_2_ and NO_X_ oxides in the densely urbanized and industrial areas of the region.

In addition, this outcome supports the findings of a study conducted in [32] that evaluated the air quality in 14 major Italian cities over the course of ten years, gathering data on PM_10_, PM_2.5_ and NO_2_ concentrations from MCUs, as well as information on public transportation and motor vehicle emissions. The two major cities in northern Italy, Turin and Milan, were found to be the most critical areas where the concentration of NO_2_ remains very high, despite a progressive decline. This is mostly because of the exhaust gas produced by motor vehicles.

In a nutshell, the proposed method, compared to ML hotspot detection methods, has the advantage of being replicable in different urban settlements; it does not require a large number of parameters and MCUs to measure the concentration of pollutants. Comparisons of the maps of the detected hotspots and critical urban areas with the results obtained in other studies have validated the accuracy of the experimental method in the study area.

On the other hand, an insufficient density of MCUs can affect the accuracy of the results since, in urbanized areas with high population densities, the presence of MCUs must be higher than in predominantly non-urbanized areas.

## 5. Conclusions

An air pollutant hotspot detection method in urban settlements during heatwaves is presented. After selecting the time frames during which heatwaves were recorded over the study area, the data relating to the concentration of the pollutants measured by MCUs during the time frames were extracted, and a daily mean value of the pollutant weighted for the duration of the heatwave was calculated. A spatial interpolation algorithm is used to obtain the distribution of the concentrations of the pollutants. Then, through a fuzzification process, the hotspots consisting of the areas where the concentration of the pollutants is highly critical for the health of the inhabitants are extracted. Finally, by analyzing the distribution of the exposed population within the hotspots, a criticality map is extracted, in which the areas of greatest criticality included in the hotspots are highlighted. The method was tested on an urban study area in the Lombardy region in Italy. The population density of those younger than 6 years and older than 74 years was considered as the value exposed to risk.

The results highlight a critical urban area that includes the metropolitan city of Milan, in line with experts’ assessments and with assessments and predictions presented in the literature.

Compared to other air pollutant hotspot detection approaches, this method has the advantage of not requiring the setting of many parameters that need to be optimized and of pollutant concentration measurements with high spatial resolution, ensuring high replicability. It represents a tool to support various decision makers, such as local administrators, air pollution monitoring and control bodies, and citizen health protection bodies.

The critical aspect of this method is that it may be inaccurate if the density of MCUs in the study area is insufficient to ensure the restitution of an accurate spatial distribution of the pollutant. This may happen in rural areas or areas with low population density, where the number of MCUs covering the study area is insufficient to provide an accurate spatial distribution of pollutants.

In the future, we intend to extend the application of the method to different types of air pollutants and to various types of population exposed to health risks; furthermore, we intend to improve the accuracy of the interpolation by considering detailed scale measurements of the concentration of pollutants carried out using mobile stations. Additionally, the further development of the research will concern the integration of the method in medium and long-term forecasting models to predict possible future scenarios of alarm for the health of citizens.

## Figures and Tables

**Table 1:** Heatwave scenarios in the period 1 June–30 September 2024.

ID Scenario	Time Frame		Number of Consecutive Days	Day with the Highest Heat Index
	Start	End		
1	28 June	30 June	3	28 June (+40 °C)
2	7 July	11 July	4	10 July (+43 °C)
3	13 July	6 August	25	19 July (+47 °C)
4	9 August	17 August	9	12 August (+52 °C)
5	20 August	1 September	13	31 August (+45 °C)

**Table 2:** Classification of the NO_2_ concentrations based on the Italian national legislative decree n° 155 of 2010. The hyphen symbol (-) means that the class does not represent a hotspot.

Range Values	Class	Hotspost
NO_2_ < 100 μg/m^3^	*Normal*	-
100 μg/m^3^ ≤ NO_2_ < 140 μg/m^3^	*To be monitored*	-
140 μg/m^3^ ≤ NO_2_ < 200 μg/m^3^	*Dangerous*	-
200 μg/m^3^ ≤ NO_2_ < 400 μg/m^3^	*Critical*	-
NO_2_ ≥ 400 μg/m^3^	*Very critical*	Hotspot

**Table 3:** Classification of disadvantaged population density.

Range Values [Inhabitants/km^2^]	Class
Density < 500	*Low*
500 ≤ Density < 2500	*Medium low*
2500 ≤ Density < 5000	*Medium*
5000 ≤ Density < 10,000	*Medium high*
Density ≥ 10,000	*High*

## Data Availability

The data presented in this study are available on request from the corresponding author.
